# Supervised machine learning including environmental factors to predict in-hospital outcomes in acute heart failure patients

**DOI:** 10.1093/ehjdh/ztae094

**Published:** 2024-12-16

**Authors:** Benjamin Sibilia, Solenn Toupin, Nabil Bouali, Jean-Baptiste Brette, Arthur Ramonatxo, Guillaume Schurtz, Kenza Hamzi, Antonin Trimaille, Emmanuel Gall, Nicolas Piliero, Alexandre Unger, Stéphane Andrieu, Trecy Gonçalves, Fabien Picard, Vincent Roule, François Roubille, Sonia Houssany-Pissot, Océane Bouchot, Victor Aboyans, Reza Rossanaly Vasram, Thomas Bochaton, Damien Logeart, Alain Cohen Solal, Jérôme Cartailler, Alexandre Mebazaa, Jean-Guillaume Dillinger, Patrick Henry, Théo Pezel

**Affiliations:** Service de Cardiologie, Université Paris Cité, Hôpital Lariboisière, Assistance Publique-Hôpitaux de Paris, 2 rue Ambroise Paré, Paris 75010, France; Inserm MASCOT—UMRS 942, University Hospital of Lariboisiere, 2 rue Ambroise Paré, Paris 75010, France; MIRACL.ai Laboratory, Multimodality Imaging for Research and Analysis Core Laboratory and Artificial Intelligence, University Hospital of Lariboisiere (AP-HP), 2 rue Ambroise Paré, Paris 75010, France; Service de Cardiologie, Université Paris Cité, Hôpital Lariboisière, Assistance Publique-Hôpitaux de Paris, 2 rue Ambroise Paré, Paris 75010, France; Inserm MASCOT—UMRS 942, University Hospital of Lariboisiere, 2 rue Ambroise Paré, Paris 75010, France; MIRACL.ai Laboratory, Multimodality Imaging for Research and Analysis Core Laboratory and Artificial Intelligence, University Hospital of Lariboisiere (AP-HP), 2 rue Ambroise Paré, Paris 75010, France; Department of Cardiology, University Hospital of Poitiers, Poitiers 86000, France; Service de Cardiologie, Centre Hospitalier de Saintonge, 11, Boulevard Ambroise-Paré, Saintes 17100, France; Cardiology Department, Rangueil University Hospital, Toulouse, France; Department of Cardiology, University Hospital of Poitiers, Poitiers 86000, France; Department of Cardiology, University Hospital of Lille, Lille, France; Service de Cardiologie, Université Paris Cité, Hôpital Lariboisière, Assistance Publique-Hôpitaux de Paris, 2 rue Ambroise Paré, Paris 75010, France; Inserm MASCOT—UMRS 942, University Hospital of Lariboisiere, 2 rue Ambroise Paré, Paris 75010, France; MIRACL.ai Laboratory, Multimodality Imaging for Research and Analysis Core Laboratory and Artificial Intelligence, University Hospital of Lariboisiere (AP-HP), 2 rue Ambroise Paré, Paris 75010, France; Department of Cardiovascular Medicine, Nouvel Hôpital Civil, Strasbourg University Hospital, Strasbourg 67000, France; Service de Cardiologie, Université Paris Cité, Hôpital Lariboisière, Assistance Publique-Hôpitaux de Paris, 2 rue Ambroise Paré, Paris 75010, France; Inserm MASCOT—UMRS 942, University Hospital of Lariboisiere, 2 rue Ambroise Paré, Paris 75010, France; MIRACL.ai Laboratory, Multimodality Imaging for Research and Analysis Core Laboratory and Artificial Intelligence, University Hospital of Lariboisiere (AP-HP), 2 rue Ambroise Paré, Paris 75010, France; Service de Cardiologie, Univ. Grenoble Alpes, CHU Grenoble Alpes, Grenoble 38000, France; Service de Cardiologie, Université Paris Cité, Hôpital Lariboisière, Assistance Publique-Hôpitaux de Paris, 2 rue Ambroise Paré, Paris 75010, France; Inserm MASCOT—UMRS 942, University Hospital of Lariboisiere, 2 rue Ambroise Paré, Paris 75010, France; MIRACL.ai Laboratory, Multimodality Imaging for Research and Analysis Core Laboratory and Artificial Intelligence, University Hospital of Lariboisiere (AP-HP), 2 rue Ambroise Paré, Paris 75010, France; Department of Cardiology, Hôpital Universitaire de Bruxelles, Hôpital Erasme, Université Libre de Bruxelles, Brussels, Belgium; Service de Cardiologie, Hôpital Henri Duffaut, Avignon 84902, France; Service de Cardiologie, Université Paris Cité, Hôpital Lariboisière, Assistance Publique-Hôpitaux de Paris, 2 rue Ambroise Paré, Paris 75010, France; Inserm MASCOT—UMRS 942, University Hospital of Lariboisiere, 2 rue Ambroise Paré, Paris 75010, France; MIRACL.ai Laboratory, Multimodality Imaging for Research and Analysis Core Laboratory and Artificial Intelligence, University Hospital of Lariboisiere (AP-HP), 2 rue Ambroise Paré, Paris 75010, France; Service de Cardiologie, Université Paris Cité, Hôpital Cochin, Paris, France; Department of Cardiology, Caen University Hospital, Caen, France; Cardiology Department, University Hospital, PhyMedExp, University of Montpellier, INSERM, CNRS, CHRU, INI-CRT, Montpellier, France; Service de Cardiologie et Médecine Aéronautique, Hôpital d'Instruction des Armées Percy, Clamart 92140, France; Service de Cardiologie, Centre Hospitalier Annecy Genevois, Epagny Metz-Tessy 74370, France; Department of Cardiology, University Hospital of Limoges, Limoges, France; Department of Cardiology, Felix-Guyon University Hospital, Saint-Denis-de-La-Reunion, France; Intensive Cardiological Care Division, Louis Pradel Hospital, Hospices Civils de Lyon, Bron, France; Service de Cardiologie, Université Paris Cité, Hôpital Lariboisière, Assistance Publique-Hôpitaux de Paris, 2 rue Ambroise Paré, Paris 75010, France; Inserm MASCOT—UMRS 942, University Hospital of Lariboisiere, 2 rue Ambroise Paré, Paris 75010, France; Service de Cardiologie, Université Paris Cité, Hôpital Lariboisière, Assistance Publique-Hôpitaux de Paris, 2 rue Ambroise Paré, Paris 75010, France; Inserm MASCOT—UMRS 942, University Hospital of Lariboisiere, 2 rue Ambroise Paré, Paris 75010, France; Inserm MASCOT—UMRS 942, University Hospital of Lariboisiere, 2 rue Ambroise Paré, Paris 75010, France; MIRACL.ai Laboratory, Multimodality Imaging for Research and Analysis Core Laboratory and Artificial Intelligence, University Hospital of Lariboisiere (AP-HP), 2 rue Ambroise Paré, Paris 75010, France; Inserm MASCOT—UMRS 942, University Hospital of Lariboisiere, 2 rue Ambroise Paré, Paris 75010, France; Service de Cardiologie, Université Paris Cité, Hôpital Lariboisière, Assistance Publique-Hôpitaux de Paris, 2 rue Ambroise Paré, Paris 75010, France; Inserm MASCOT—UMRS 942, University Hospital of Lariboisiere, 2 rue Ambroise Paré, Paris 75010, France; MIRACL.ai Laboratory, Multimodality Imaging for Research and Analysis Core Laboratory and Artificial Intelligence, University Hospital of Lariboisiere (AP-HP), 2 rue Ambroise Paré, Paris 75010, France; Service de Cardiologie, Université Paris Cité, Hôpital Lariboisière, Assistance Publique-Hôpitaux de Paris, 2 rue Ambroise Paré, Paris 75010, France; Inserm MASCOT—UMRS 942, University Hospital of Lariboisiere, 2 rue Ambroise Paré, Paris 75010, France; MIRACL.ai Laboratory, Multimodality Imaging for Research and Analysis Core Laboratory and Artificial Intelligence, University Hospital of Lariboisiere (AP-HP), 2 rue Ambroise Paré, Paris 75010, France; Service de Cardiologie, Université Paris Cité, Hôpital Lariboisière, Assistance Publique-Hôpitaux de Paris, 2 rue Ambroise Paré, Paris 75010, France; Inserm MASCOT—UMRS 942, University Hospital of Lariboisiere, 2 rue Ambroise Paré, Paris 75010, France; MIRACL.ai Laboratory, Multimodality Imaging for Research and Analysis Core Laboratory and Artificial Intelligence, University Hospital of Lariboisiere (AP-HP), 2 rue Ambroise Paré, Paris 75010, France

**Keywords:** Acute heart failure, Environmental factors, Carbon monoxide, Recreational drugs, Machine learning, Intensive cardiac care unit

## Abstract

**Aims:**

While few traditional scores are available for risk stratification of patients hospitalized for acute heart failure (AHF), the potential benefit of machine learning (ML) is not well established. We aimed to assess the feasibility and accuracy of a supervised ML model including environmental factors to predict in-hospital major adverse events (MAEs) in patients hospitalized for AHF.

**Methods and results:**

In April 2021, a French national prospective multicentre study included all consecutive patients hospitalized in intensive cardiac care unit. Patients admitted for AHF were included in the analyses. A ML model involving automated feature selection by least absolute shrinkage and selection operator (LASSO) and model building with a random forest (RF) algorithm was developed. The primary composite outcome was in-hospital MAE defined by death, resuscitated cardiac arrest, or cardiogenic shock requiring assistance. Among 459 patients included (age 68 ± 14 years, 68% male), 47 experienced in-hospital MAE (10.2%). Seven variables were selected by LASSO for predicting MAE in the training data set (*n* = 322): mean arterial pressure, ischaemic aetiology, sub-aortic velocity time integral, E/e′, tricuspid annular plane systolic excursion, recreational drug use, and exhaled carbon monoxide level. The RF model showed the best performance compared with other evaluated models [area under the receiver operating curve (AUROC) = 0.82, 95% confidence interval (CI) (0.78–0.86); precision-recall area under the curve = 0.48, 95% CI (0.42–0.5), *F*1 score = 0.56). Our ML model exhibited a higher AUROC compared with an existing score for the prediction of MAE (AUROC for our ML model: 0.82 vs. ACUTE HF score: 0.57; *P* < 0.001).

**Conclusion:**

Our ML model including in particular environmental variables exhibited a better performance than traditional statistical methods to predict in-hospital outcomes in patients admitted for AHF.

**Study registration:**

ClinicalTrials.gov identifier: NCT05063097.

## Graphical Abstract

**Graphical Abstract:**
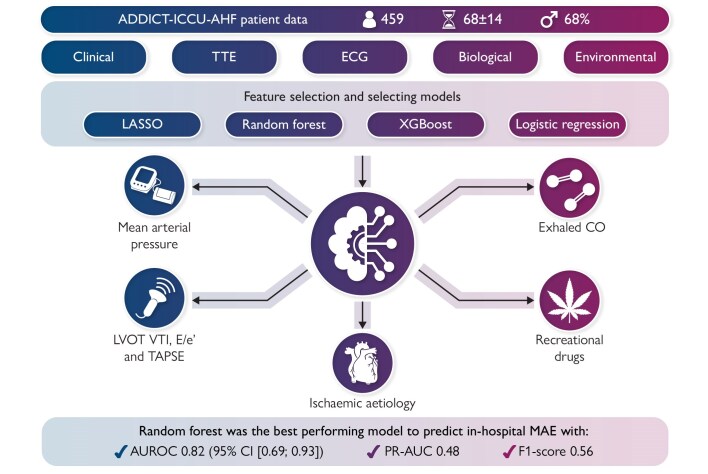
Prediction models using machine learning including environmental factors to assess the risk of in-hospital major adverse events in patients hospitalized for acute heart failure. ADDICT-ICCU, Addiction in intensive cardiac care unit; AHF, acute heart failure; CO, carbon monoxide; LASSO, least absolute shrinkage and selection operator; MAE, major adverse event; PR, precision-recall; TAPSE, tricuspid annular plane systolic excursion; TTE, transthoracic echocardiogram; LVOT VTI, left ventricular outflow tract velocity–time integral; XGBoost, extreme gradient boosting.

## Introduction

Acute heart failure (AHF) continues to be the main cause for hospital admissions among patients over the age of 65 years and is responsible for a mortality exceeding 25%,^1^ including an 8% mortality rate within hospitals at the 30-day mark.^2^ It is therefore crucial to offer a high-performance prognostic stratification tool to identify these patients at risk of in-hospital poor outcomes. Beyond the clinical, biological, and echocardiographic parameters well established as prognostic factors,^3,4^ several recent studies have shown a role for environmental factors in the in-hospital prognosis of these AHF patients. Indeed, recent studies reported that recreational drug use could be a strong prognosticator of in-hospital outcomes due to multifactorial acute cardiotoxicity.^5–7^ Indeed, our working group has recently shown that recent consumption of recreational drugs in AHF patients was independently associated with a risk of in-hospital major adverse event (MAE) multiplied by more than seven compared with non-users.^6^ Another recent subanalysis showed that the level of exhaled CO, as a biomarker of air pollution in the patient's lungs, was independently associated with a higher risk of in-hospital mortality in AHF patients.^8^ Furthermore, a large meta-analysis emphasized the impact of air pollution, including carbon monoxide (CO) due to active and passive smoking, as an aggravating factor of AHF.^9^ However, until now, no tool is available to take into account all of these variables simultaneously.

Machine learning (ML) methods can recognize patterns in large data sets with a multiplicity of variables and can be used for improving risk stratification. Recently, ML techniques, using supervised or unsupervised approaches, have emerged as highly effective methods for prognostic prediction and decision-making in several cardiovascular diseases.^10,11^ Compared with unsupervised ML, a supervised ML method overcomes the main limitations of traditional methods with the problems of collinearity, inadequate model complexity, and violation of rigid assumptions. In addition, the strength of supervised ML is its ability to approximate non-linear relationships between hundreds of variables. Although of potential interest, supervised ML techniques using all this spectrum of variables have not been evaluated for risk stratification in AHF patients.

Therefore, the aim of this study was to investigate the feasibility and accuracy of a supervised ML model using clinical, biological, environmental, and echocardiographic data to predict in-hospital MAE in a consecutive cohort of patients hospitalized for AHF.

## Methods

### Study population

**Figure 1:**
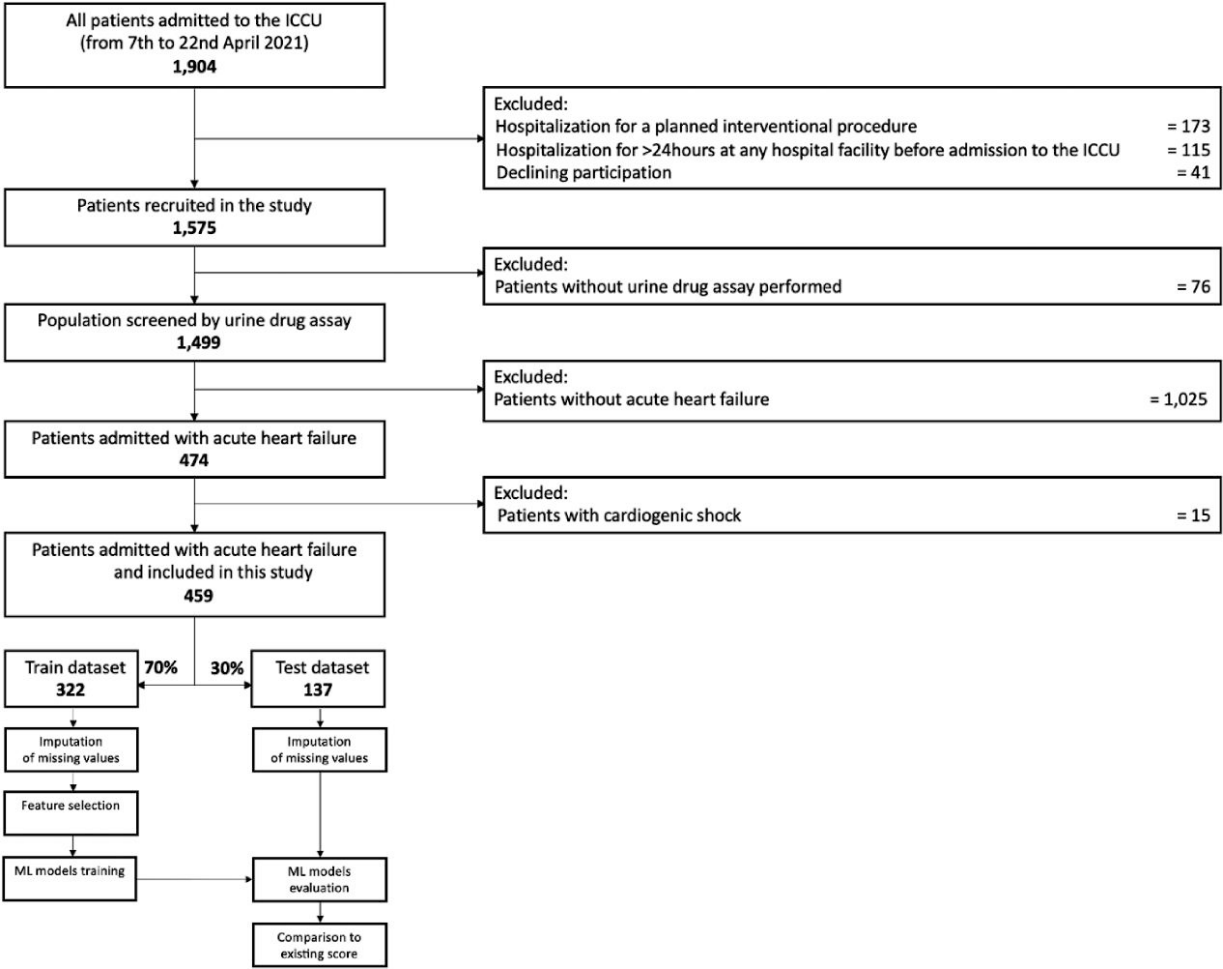
Flowchart of the study population. ICCU, intensive cardiac care unit; ML, machine learning.

All details of the Addiction in Intensive Cardiac Care Units (ADDICT-ICCU) study design have been published previously.^12^ Briefly, this is a multicentre, prospective, observational study of all consecutive patients aged ≥18 years admitted to intensive cardiac care units (ICCUs) over 2 weeks in April 2021 at 39 centres across France (including all diagnoses). The main exclusion criterion was hospitalization for either a planned interventional procedure or more than 24 h at any hospital facility before admission to the ICCU. This was to prevent the risk of obtaining a negative urine drug assay in patients with recreational drug consumption more than 24 h prior to admission. The methodology of the baseline characteristics collection is detailed in Supplementary material online, *Supplement S1*. In this study, we decided to focus the analysis on patients admitted for AHF defined by symptoms and/or signs of heart failure with evidence of diastolic or systolic dysfunction by echocardiography and elevated levels of natriuretic peptide [B-type natriuretic peptide (BNP) > 35 pg/mL and/or N-terminal prohormone of BNP (NT-proBNP) > 125 pg/mL]^3^ (*Figure 1*). The main admission diagnosis was adjudicated by two independent experts at the end of the hospitalization following the current guidelines (see Supplementary material online, *Supplement S2*). The investigation conforms with the principles outlined in the Declaration of Helsinki. All patients had a written informed consent obtained at enrolment and were informed of the urine drug assay and exhaled CO measurement. This study has been approved by the Ethics Committee (Committee for the Protection of Human Subjects, Ile de France-7, France) and registered on the ClinicalTrials.gov website under the number NCT05063097.

### Primary outcome

The primary outcome was in-hospital MAE defined as a composite of all-cause mortality, cardiogenic shock (requiring medical or mechanical haemodynamic support) and resuscitated cardiac arrest (severe ventricular arrhythmia requiring defibrillation or anti-arrhythmic agents). An independent committee of experts was in charge of reviewing anonymized medical documents according to standardized definitions.^13^

### Baseline characteristics collection

The database records of the AHF population comprise baseline data that include clinical, demographic, reason for hospitalization, list of medications especially cardiovascular medications at admission, and history of cardiovascular disease. Blood pressure was measured non-invasively. Biological data were collected systematically upon admission, including haemoglobin, serum potassium, creatinine, the peak of troponin (hsTNI), the NT-proBNP or BNP. Standardized transthoracic echocardiography was performed systematically within the first 24 h of admission for all patients by a cardiologist and reviewed by a senior.

Regarding environmental factors, the presence of recreational drugs was determined through urine analysis using a dedicated drug assay (NarcoCheck®, Kappa City Biotech SAS, Montluçon, France) within 2 h of admission to the ICCU with excellent performance (the sensitivity and specificity of the urine drug assay were 91.7% and 97.7%, respectively).^6^ The following recreational drugs will be screened for all patients: cannabinoids (tetrahydrocannabinol), including cannabis and hashish; cocaine and metabolites, including cocaine and crack; amphetamines; 3,4-methylenedioxy-methylamphetamine (or ecstasy); and heroin and other opioids. Finally, a standardized exhaled CO measurement was systematically performed with a CO-Check Pro device (Micro Direct Diagnostics Ltd, UK) immediately on arrival in ICCU.

## Machine learning

Fourteen clinical, four biological, three environmental, and seven echocardiographic parameters were available (detailed list provided in Supplementary material online, *Supplement S3*). Machine learning involved feature selection by least absolute shrinkage and selection operator (LASSO) algorithm and model building with random forest algorithm following guidelines for ML.^14^

### Feature selection

**Figure 2:**
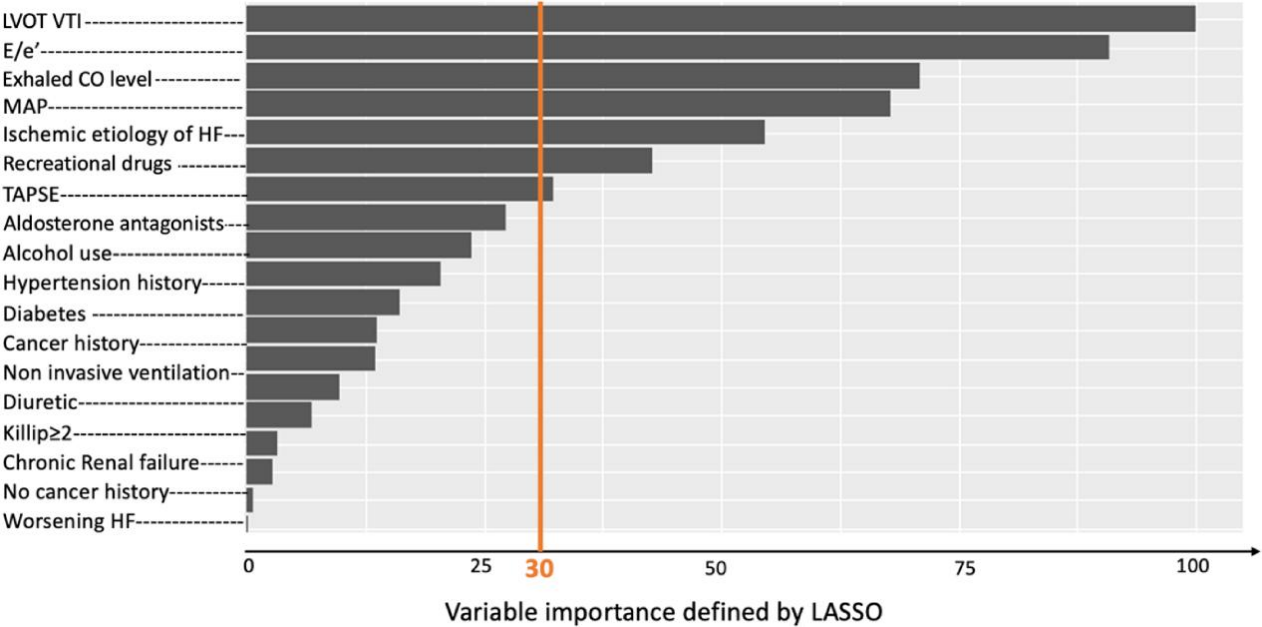
Feature importance selected by least absolute shrinkage and selection operator. Least absolute shrinkage and selection operator was used to evaluate the worth of each variable by measuring the log-rank–based variable importance with respect to the outcome and then to rank the attributes by their individual evaluations (top to bottom). Only attributes resulting in log-rank based variable importance > 30 (above the vertical line) were subsequently used for the model building. CO, carbon monoxide; HF, heart failure; LASSO, least absolute shrinkage and selection operator; LVOT VTI, left ventricular outflow tract velocity–time integral; MAP, mean arterial; TAPSE, tricuspid annular plane systolic excursion.

After the imputation of missing values by the *K*-nearest neighbours with a value of *K* = 5, feature selection was performed using LASSO (*Figure 2*), which is a penalized logistic regression that reduces the number of variables (glmnet R package).^15^ The regularization parameter *λ* controls the strength of the shrinkage to get the best compromise between prediction performance and interpretability. The best *λ* was found using 10-fold cross-validation and the ‘1 standard error rule’, for which the area under the receiver operating curve (AUROC) of the most parsimonious model is no more than 1 standard error above the AUROC of the best model.^16^

### Model building

**Figure 3:**
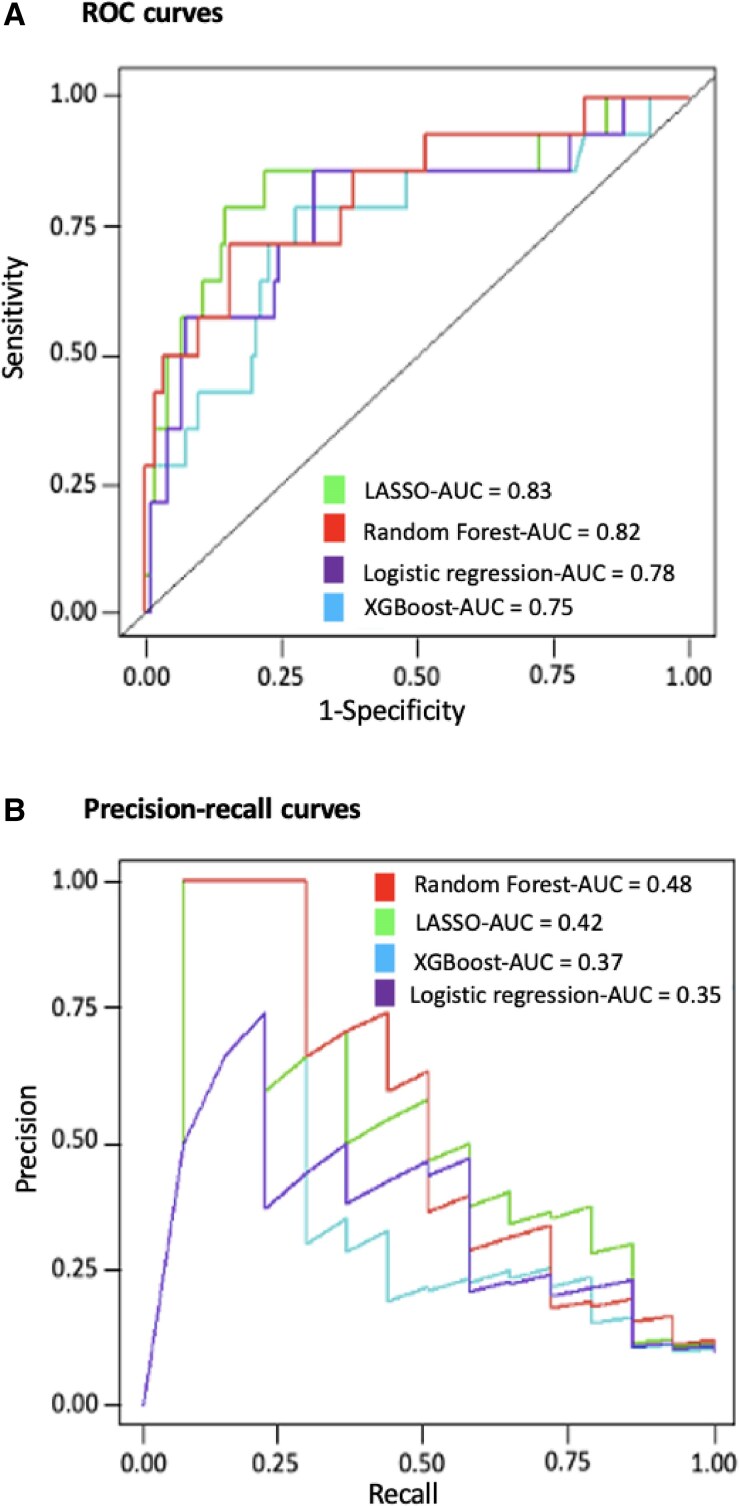
Performance of machine learning models for prediction of in-hospital major adverse event. Receiver operating characteristic (area under the receiver operating curve, *A*) and precision-recall curves (precision-recall area under the curve, *B*) for prediction of in-hospital MAE were used to compare least absolute shrinkage and selection operator, random forest, logistic regression, and extreme gradient boosting, developed using the set of variables selected by least absolute shrinkage and selection operator. AUC, area under the curve; LASSO, least absolute shrinkage and selection operator; ROC, receiver operating curve; XGBoost, extreme gradient boosting.

To build the prediction model based on the training cohort, we compared four modelling strategies with logistic regression, random forest, extreme gradient boosting (XGBoost) model, and LASSO. To assess the performance of these algorithms, we used several metrics: AUROC, precision-recall area under the curve (PRAUC), *F*1 score, sensitivity, specificity, positive and negative prediction value, accuracy, balanced accuracy, Cohen’s kappa, Brier score, and McNemar test *P*-value. Due to the low occurrence of events and the imbalanced nature of the data, the *F*1 score and the precision-recall curve were preferred to determine the best model to minimize the overall misclassification cost and mitigate the problem of imbalanced data. For the prediction of in-hospital MAE, the best model was the ML model using random forest (*Figure 3*). Random forest corresponds to a method based on decision trees, where each tree is trained on a random sample from the data set. All the results presented are shown using only the testing cohort for validation.

For model development, patients hospitalized for AHF in the ADDICT-ICCU study were randomly partitioned into training (70%) and validation (30%) cohorts (see Supplementary material online, *Supplement S4*). We used a 10-fold cross-validation process to tune the model hyperparameters using grid search.

### Statistical analysis

Continuous data will be reported as mean ± standard deviation for normally distributed data or as medians and interquartile range for non-normally distributed data. Categorical data will be reported as counts and percentages. Between-group comparisons will be performed using the Student’s *t*-test or Mann–Whitney test for continuous variables and the *χ*^2^ or Fisher’s exact test for categorical variables, as appropriate.

Model discrimination was measured using the AUROC and its 95% confidence intervals (CIs). In addition, the positive predictive value (or precision) and the sensitivity (recall) across all possible risk thresholds for predicting in-hospital MAE were plotted using precision-recall curves and their area under the curve. The PRAUC, unlike the AUROC, is not affected by the number of true-negative results. In data sets with small event rates and therefore a large expected true-negative rate, such as the current study, the PRAUC is better for comparing different models. For both the AUROC and PRAUC, values closer to 1 correspond to more accurate models. DeLong's test was used to compare the AUROC of each model. We also calculated the *F*1 score, sensitivity, specificity, positive predictive value, and negative predictive value. In addition, we calculated a Brier score for each model as a measure of model accuracy.

Model calibration was measured using (i) the Hosmer–Lemeshow goodness-of-fit test; (ii) the reliability component of the Brier score; and (iii) graphically with plots showing the observed and predicted proportion of events, grouped by level of risk.

A two-tailed *P*-value < 0.05 will be considered statistically significant. All data will be analysed using R software, version 4.0.3 (R Project for Statistical Computing, R Foundation, Vienna, Austria).

## Results

### Study population

Detailed flowchart of the study is depicted in *Figure 1*. Among the whole population of the study, 459 patients were admitted for AHF (68% male, mean age 68 ± 14 years), 46% had ischaemic cardiomyopathy and 53% with LVEF value < 50%. All characteristics of the population are described in *Table 1*. Concerning the main admission diagnosis associated with AHF, 135 (29%) patients had acute coronary syndromes, 39 (9%) had severe conduction/arrhythmia abnormalities, 29 (6%) had pulmonary embolism, 20 (4%) had acute myocarditis, 9 (2%) had Takotsubo syndrome, 6 (1%) had spontaneous coronary dissection, 24 (5%) had other cardiovascular diagnoses, and 197 (44%) had isolated AHF.

**Table 1:** Baseline characteristics according to the occurrence of in-hospital major adverse events (*N* = 459)

	All patients (*n* = 459)	Patients without MAE (*n* = 412)	Patients with MAE (*n* = 47)	*P*-value
**Demographic data**				
Age, years	68 ± 14	68 ± 14	70 ± 15	0.37
Males, *n* (%)	311 (67.8%)	276 (67.0%)	35 (74.5%)	0.30
Body mass index, kg/m²	28 ± 6	28 ± 7	27 ± 5	0.32
**Cardiovascular risk factors**, *n* (%)				
Diabetes mellitus	131 (28.5%)	112 (27.2%)	19 (40.4%)	0.06
Hypertension	287 (62.5%)	255 (61.9%)	32 (68.1%)	0.41
Dyslipidaemia	204 (44.4%)	183 (44.4%)	21 (44.7%)	0.97
Current or previous smoking	285 (63.1%)	255 (63.0%)	30 (63.8%)	0.91
Chronic obstructive pulmonary disease	38 (8.3%)	32 (7.8%)	6 (12.8%)	0.26
**History of CV disease, *n* (%)**				
Known CAD	107 (23.3%)	93 (22.6%)	14 (29.8%)	0.27
Chronic renal failure	96 (20.9%)	85 (20.6%)	11 (23.4%)	0.66
History of HF hospitalization	65 (14.2%)	58 (14.1%)	7 (14.9%)	0.88
**Clinical parameters at admission**				
Mean arterial pressure, mmHg	98 ± 21	100 ± 20	84 ± 19	<0.001
Heart rate, b.p.m.	91 ± 27	90 ± 27	96 ± 28	0.17
Oxygen saturation, %	96.3 ± 3.6	96.4 ± 3.6	95.9 ± 3.5	0.22
Killip class				
I	216 (47.1%)	194 (47.1%)	22 (46.8%)	0.97
II	169 (36.8%)	151 (36.7%)	18 (38.3%)	0.82
III	74 (16.1%)	67 (16.3%)	7 (14.9%)	0.81
Main admission diagnosis		412	47	
Isolated AHF	197 (43%)	185 (45%)	12 (26%)	<0.001
Acute coronary syndrome	135 (29%)	117 (29%)	18 (38%)	<0.001
Conduction abnormalities/arrhythmia	39 (9%)	37 (9%)	2 (4%)	0.12
Pulmonary embolism	29 (6%)	25 (6%)	4 (9%)	0.42
Acute myocarditis	20 (4%)	16 (4%)	4 (7%)	0.09
Takotsubo syndrome	9 (2%)	8 (2%)	1 (2%)	0.87
Coronary dissection	6 (1%)	4 (1%)	2 (4%)	0.67
Other CV diagnoses	24 (5%)	17 (5%)	4 (8%)	0.12
**Laboratory results**				
Haemoglobin, g/dL	12.9 ± 2.2	12.9 ± 2.2	12.4 ± 2.4	0.18
Creatinemia, µmol/L	118 ± 91	116 ± 91	128 ± 83	0.15
High-sensitivity cardiac troponin peak, Ul/L	354 ± 1906	335 ± 1979	516 ± 1079	<0.001
NT-proBNP, pg/mL	13 960 ± 23 843	13 815 ± 24 401	15 233 ± 18 396	0.145
**Echocardiography data**				
LA dilatation ≥ 32 mL/m², *n* (%)	144 (31.4%)	123 (29.9%)	21 (44.7%)	0.038
Mitral E/A ratio	1.32 ± 0.71	1.30 ± 0.72	1.49 ± 0.55	<0.001
Mitral E/e′ ratio	10.7 ± 4.2	10.3 ± 3.8	13.9 ± 5.6	<0.001
LVEF, %	45 ± 16	46 ± 16	37 ± 17	<0.001
LVEDV, mL/m²	124 ± 57	122 ± 56	135 ± 57	0.089
LVOT VTI, cm	17.7 ± 5.9	18.1 ± 5.7	14.2 ± 5.8	<0.001
sPAP, mmHg	31 ± 15	31 ± 15	34 ± 14	0.048
RV dilation, *n* (%)	63 (13.7%)	58 (14.1%)	5 (10.6%)	0.52
TAPSE, mm	19.2 ± 4.8	19.4 ± 4.7	16.6 ± 4.8	<0.001
**Heart failure data**				
Worsening heart failure	95 (20.7%)	86 (20.9%)	9 (19.1%)	0.78
Ischaemic aetiology of heart failure	212 (46.2%)	180 (43.7%)	32 (68.1%)	0.001
Type of heart failure				0.011
HFrEF	155 (33.8%)	131 (31.8%)	24 (51.1%)	
HFmEF	87 (19.0%)	77 (18.7%)	10 (21.3%)	
HFpEF	217 (47.3%)	204 (49.5%)	13 (27.7%)	
**Previous treatment**				
Aldosterone antagonists	44 (9.6%)	35 (8.5%)	9 (19.1%)	0.032
ACEI/ARB, or Entresto	219 (47.7%)	195 (47.3%)	24 (51.1%)	0.63
Diuretics	136 (29.6%)	117 (28.4%)	19 (40.4%)	0.09
**Environmental factors**				
Alcohol consumption	230 (50.1%)	210 (51.0%)	20 (42.6%)	0.27
CO, ppm	4.5 ± 5.1	4.2 ± 4.4	7.8 ± 8.5	0.026
Recreational drug use, *n* (%)	42 (9.2%)	30 (7.3%)	12 (25.5%)	<0.001

Values are *n* (%), mean ± SD, or median (interquartile range).

ACEI/ARB, angiotensin-converting enzyme inhibitor/angiotensin receptor blocker; CAD, coronary artery disease; CO, carbone monoxide; CV, cardiovascular; HF, heart failure; HFrEF, heart failure reduced ejection fraction; HFmEF, heart failure mild ejection fraction; HFpEF, heart failure preserved ejection fraction; LA, left atrium; LVEF, left ventricular ejection fraction; LVEDV, left ventricular end diastolic volume; LVOT VTI, left ventricular outflow tract velocity–time integral; MAE, major adverse event; NT-proBNP, N-terminal prohormone of B-type natriuretic peptide; RV, right ventricular; sPAP, systolic pulmonary artery pressure; TAPSE, tricuspid annular plane systolic excursion

During a median duration of hospitalization of 7 days (interquartile range 5–12 days) in the ICCU, 47 patients (10.2%) experienced in-hospital MAE, including 18 (3.9%) in-hospital deaths, 8 (1.7%) resuscitated cardiac arrests, and 21 (4.6%) cardiogenic shocks requiring medical and/or mechanical haemodynamic support. Patients who experienced in-hospital MAE had a lower mean arterial pressure, a higher cardiac troponin level, a lower LVEF value, and tricuspid annular plane systolic excursion (TAPSE) value compared with patients without in-hospital MAE (all *P* < 0.001).

### Feature selection

Using the LASSO feature selection method on the training cohort with the best regularization parameter *λ* (see Supplementary material online, *Supplement S5*), seven of the available variables were selected for the ML model (two clinical, two environmental, and three echocardiographic) in descending order of importance: left ventricular outflow tract velocity–time integral (LVOT VTI), peak E/e′ ratio, exhaled CO level, mean arterial pressure, ischaemic AHF aetiology, recreational drug use, and TAPSE (*Figure 2*).

### Model building

The ML model using random forest exhibited the best global performance for the prediction of in-hospital MAE in the testing cohort compared with LASSO, stepwise regression, and XGBoost using all the metrics available (*Table 2*).

**Table 2:** Performance of some evaluated machine learning models for predicting in-hospital major adverse event

Evaluation metrics	Logistic regression	LASSO	Random forest	XGBoost
AUROC	0.78 (0.74–0.82)	0.83 (0.79–0.87)	0.82 (0.78–0.86)	0.75 (0.70–0.80)
PRAUC	0.35 (0.29–0.41)	0.42 (0.38–0.46)	0.48 (0.42–0.54)	0.37 (0.26–0.44)
Accuracy	0.88	0.90	0.92	0.87
Cohen’s kappa	0.21	0.36	0.52	0.28
Sensitivity	0.36	0.36	0.50	0.36
Specificity	0.93	0.96	0.97	0.93
Precision	0.38	0.50	0.64	0.37
*F*1 score	0.37	0.42	0.56	0.36
Brier score	0.08	0.07	0.07	0.08

Evaluation of the performance of logistic regression, LASSO, random forest, and XGBoost.

AUC, area under the curve; LASSO, least absolute shrinkage and selection operator; PRAUC, precision-recall area under the curve; ROC, receiver operating characteristic; XGBoost, extreme gradient boosting.

### Model performance compared with traditional methods and existing score

**Figure 4:**
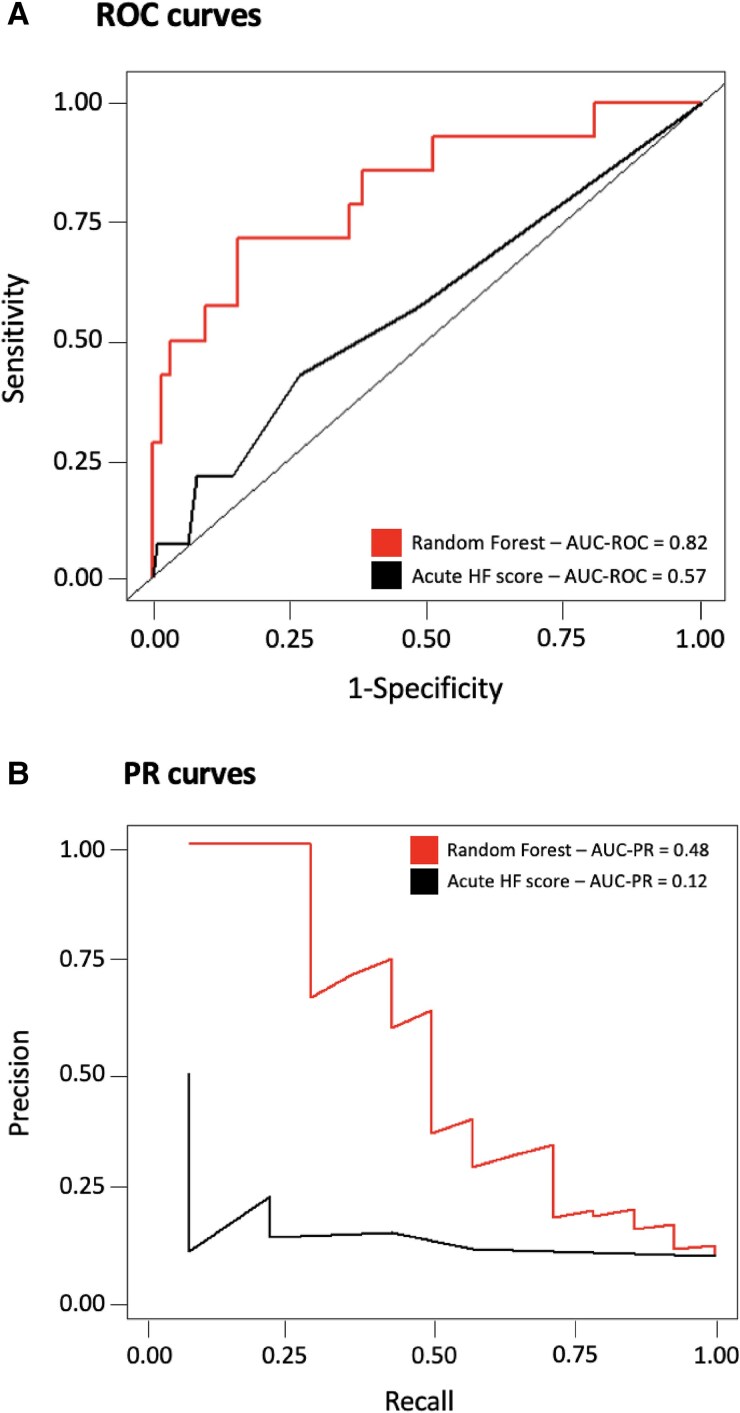
Comparison of our model with ACUTE HF score. Receiver operating characteristic (area under the receiver operating curve, *A*) and precision-recall curves (precision-recall area under the curve, *B*) for prediction of in-hospital major adverse event were used to compare our machine learning model to the ACUTE HF score. AUC, area under the curve; PR, precision-recall; ROC, receiver operating curve.

The ML model showed a better accuracy to predict in-hospital MAE compared with a logistic regression model in the testing cohort [AUROC: 0.82, 95% CI (0.78–0.86) vs. 0.78, 95% CI (0.74–0.82); PRAUC: 0.48, 95% CI (0.42–0.54) vs. 0.35, 95% CI (0.29–0.41); *Figure 3*]. The proposed ML model exhibited the best performance for the prediction of in-hospital MAE compared with the ACUTE HF score,^17^ with a higher AUROC (0.82 vs. 0.57) and PRAUC (0.48 vs. 0.12; *Figure 4*). The performances of the ML model were globally similar in women and men (see Supplementary material online, *Supplement S6*). As sensitivity analysis, our ML model exhibited the best performance for the prediction of in-hospital all-cause death compared with traditional statistical method with a higher AUROC (0.81 vs. 0.76) and PRAUC (0.46 vs. 0.32; Supplementary material online, *Supplement S7*).

### Calibration of our machine learning model

The Hosmer–Lemeshow goodness-of-fit test showed no significant difference between the observed and predicted proportions of in-hospital MAE (*P* = 0.91) in the testing cohort, in line with the Brier score of 0.07. All these findings indicated a good calibration of the proposed ML model across all risk levels (see Supplementary material online, *Supplement S8*).

## Discussion

In a prospective multicentre cohort study of consecutive patients hospitalized for AHF in ICCU, we built a ML model to predict in-hospital outcomes (*Graphical Abstract*). This ML model was based on few simple clinical, environmental, and echocardiographic variables: mean arterial pressure, ischaemic AHF aetiology, recreational drug use, exhaled CO level, LVOT VTI, peak E/e′ ratio, and TAPSE. The main findings are as follows: (i) the ML model exhibited a better performance than traditional statistical methods; (ii) the ML model outperformed existing scores as ACUTE HF score; (iii) its calibration was excellent; and (iv) among all the variables available, the feature selection using ML highlighted the importance of environmental data as recreational drug use and exhaled CO level. The study presents the first large-scale investigation of ML for prognostic risk evaluation using a wide spectrum of data ranging from clinical to echocardiography through environmental data. These findings suggest that ML and environmental factors could have an important role for risk stratification and decision-making in patients with AHF.

In line with prior large cohorts with a rate of in-hospital outcomes between 5 and 10%,^2,18,19^ we reported a rate of in-hospital MAE of 10.2% and in-hospital death of 1.7%. In our cohort, MAE was more driven by cardiogenic shock than all-cause in-hospital mortality and resuscitated cardiac arrest events.

Regarding the environmental factors selected by ML, exhaled CO level was one of the highest ranked features for risk prediction. It has been previously described as a measure of particular interest for the study of active and passive smoking exposure as an important marker of air pollution. An ancillary study of patients from the Framingham cohort showed an association between exhaled CO level and the occurrence of cardiovascular outcomes regardless of the patient's smoking status.^20^ This suggests that exhaled CO level could be an interesting marker for assessing the cardiovascular impact of air pollution. Indeed, an important meta-analysis showed a strong association between daily increases in gaseous air pollutants (particulate matter, CO, sulfur dioxide, nitrogen dioxide, and ozone) and the occurrence of severe HF outcomes.^9^ Although more studies from developing nations are required, air pollution is a pervasive public health issue with major cardiovascular and health economic burden, and it should remain a key target for global health policy. In line with these results, our working group has very recently shown that elevated CO level is independently associated with a 10-fold increase in in-hospital MAE and six-fold 1-year mortality in patients hospitalized for acute cardiac events.^8^ All these findings are also supported by Tun *et al*.,^21^ who showed that increased levels of exhaled CO were associated with an adverse cardiovascular biomarker profile and a higher risk of AHF, especially with reduced LVEF.

The second environmental factor selected by ML was recreational drug use. This finding is in line with a prior analysis from ADDICT-ICCU study showing that recreational drugs use is strongly associated with the occurrence of in-hospital outcomes with an incremental prognostic value above traditional risk factors.^6^ These findings can be explained by several types of sympathomimetic effects of recreational drugs, which can increase heart rate, blood pressure, temperature, and consequently myocardial oxygen demand.^22^

Two clinical variables were selected by ML with mean blood pressure and ischaemic aetiology of HF. Blood pressure is a well-known feature present in most of AHF scores.^23,24^ In the current study, we preferred the mean blood pressure than systolic blood pressure because it includes the effect of the diastolic value, which can reflect different situations as the aortic regurgitation or vasoplegia. Knowing that ischaemic aetiology is also well described as prognosticator for HF outcomes, two scores already published have also selected this feature as a prognosis factor.^19,25^

Beyond the traditional clinical data, the current study emphasized the importance of echocardiographic parameters at baseline in ICCU for risk stratification in AHF patients. Indeed, among the seven parameters selected by ML, three were echocardiographic variables with LVOT VTI, peak E/e′ ratio, and TAPSE, which are mean full in the day-to-day practice and can be performed by any cardiologist. Several studies have shown that left ventricular dysfunction with a drop in cardiac output,^3,4^ assessed by the LVOT VTI, was associated with a poor prognosis in a wide spectrum of acute cardiovascular diseases. Right ventricular dysfunction defined using TAPSE^26,27^ and left ventricular filling pressure assessed by the peak E/e′ ratio have also been described as two strong prognosticators in the acute setting of AHF.^2,3,28^ Consistently, these three echocardiographic parameters have a central place in the last European Society of Cardiology guidelines.^3,4^

Finally, our ML model showed the best accuracy to predict in-hospital outcomes compared with traditional methods and existing score assessed of AHF patients in ICCU. In particular, its performance outstripped ACUTE HF score.^17^ Up to date, no specific ML model including clinical, biological, environmental, and echocardiographic data has been built.

### Study limitations

First, the sample size of patients analysed was decreased due to the split between training and testing cohorts. In addition, the exclusion of eight centres to build an external validation cohort also lowered the sample size. Nevertheless, as there are no other studies on the AHF population that have measured exhaled CO and recreational drug use, the results necessarily had to be validated on a part of the ADDICT-ICCU study. This lack of external validation from another country is an important limitation of the application of these findings, and we need to have further studies to validate them in other countries. Second, even if recreational drugs use^6^ and high exhaled CO level have recently been reported as increasing cardiovascular events^8^ and had pathophysiological background on cardiovascular system, their screenings are not available in daily practice, hence a limited translation in practice. Indeed, exhaled CO level and recreational drug detection are not routinely assessed in all centres worldwide. Even if the echocardiographic findings of this study are important, we must acknowledge that a complete echocardiography including LVEF as collected in this study within 24 h after admission in ICCU is not systematically performed in clinical routine. Third, missing values always represent a challenge in medical research. In line with the literature,^29^ we imputed missing data using with K-Nearest Neighbor algorithm for all data with a missing data rate < 40%. Fourth, some ICCU scores could not be calculated due to missing biological collected data. The only score we could compare with was ACUTE HF score,^17^ and results may be interpreted with caution. The SCAI shock score was not available in this study. Finally, this study assessed only the performance of ML, but not its practical implementation. Additional parameters would have been of interest, especially renal function (not only creatininemia, C-reactive protein, intake of some drugs…). Once validated in clinical routine, further randomized clinical trials based upon ML risk stratification using environmental data should be considered.

## Conclusion

In a large multicentre registry of consecutive patients hospitalized for AHF in ICCU, a ML model using seven clinical, environmental—including exhaled CO level and recreational drugs use—and echocardiographic variables was an accurate method for predicting in-hospital MAE. This ML model exhibited a higher prognostic value to predict in-hospital MAE than all statistical methods or existing score.

## Lead author biography


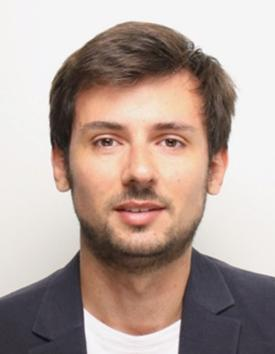


Dr Benjamin Sibilia is a cardiologist at Lariboisière-Saint Louis Hospital, Paris, since 2023, and is affiliated with the University of Paris-Cité. He holds a Master’s degree in Methodology and Statistics in Medical Research. His clinical and research interests focus on heart failure, cardiovascular prevention, and cardio-oncology. He is actively involved in the MIRACL.ai platform (Multimodality Imaging for Research and Analysis Core Laboratory and Artificial Intelligence, AP-HP, Paris, France), a cutting-edge platform that integrates academic research, artificial intelligence with machine learning projects, and industry to advance diagnostic and prognostic tools in cardiology.

## Supplementary Material

ztae094_Supplementary_Data

## Data Availability

Data are available upon reasonable request.
