# Supplementary material for: Supervised machine learning including environmental factors to predict in-hospital outcomes in acute heart failure patients
Source: Eur Heart J Digit Health. 2024 Dec 16;6(2):190–9. doi: 10.1093/ehjdh/ztae094 (PMC11914725; doi:10.1093/ehjdh/ztae094)
Supplement: ztae094_Supplementary_Data [file ztae094_supplementary_data.docx]

**SUPPLEMENTAL MATERIAL**

**eSupplement 1.** Baseline and in-hospital characteristics collection.

**eSupplement 2.** Definition of main admission diagnosis.

**eSupplement 3.** Variables used in the feature selection algorithm.

**eSupplement 4.** Baseline characteristics of the training and the testing cohorts (N=459).

**eSupplement 5.** Feature selection by LASSO

**eSupplement 6.** Evaluation of the Random Forest performances according to the gender.

**eSupplement 7.** Performance of machine learning models for predicting all-cause death.

**eSupplement 8.** Calibration curve for the ML-model

**eReferences.**

**eSupplement 1. Baseline and in-hospital characteristics collection**

***Baseline characteristics***

- Baseline data included the date of birth, gender, height, weight, temperature, systolic and diastolic blood pressures, heart rate, Glasgow score, Killip class, oxygen saturation and ventilation mode, presence of symptoms, list of medications at admission, history of cardiovascular disease, psychiatric illness or other significant clinical histories and main admission diagnosis.
- Regarding the drug addiction surveys, we assessed: i) declaration of psychoactive drug use (cannabinoids, cocaine, amphetamines, MDMA, heroin or other opioids); ii) smoking history (daily or nondaily smoker, cigarettes smoked per day by daily smokers, age started smoking, years smoked and e-cigarette use); iii) declaration of alcohol use.
- Of note, history of cardiovascular disease (CVD) was defined by the presence of: known MI, previous PCI, previous CABG, peripheral atheroma with revascularization, stroke, history of heart failure, history of atrial fibrillation, history of surgery for valvular heart disease, pacemaker or ICD, and cardiomyopathies.

***Hospital clinical characteristics***

- Electrocardiogram (ECG) and transthoracic echocardiography (TTE) with left ventricular ejection fraction (LVEF) were performed systematically within the first 24 hours of admission for all patients. Need for revascularization [percutaneous coronary intervention (PCI) or coronary artery bypass surgery (CABG)], ventricular arrhythmia (sustained ventricular tachycardia or fibrillation), cardiogenic shock, need for hemodynamic support, and urgent repeat revascularization were recorded.
- Laboratory results were also collected systematically upon admission, including hemoglobin, potassium, creatinine, the maximum peak of troponin (hsTNI), the N-terminal prohormone of B-type natriuretic peptide (NT-proBNP) or B-type natriuretic peptide (BNP).
- All the diagnostic procedures of cardiovascular imaging or invasive angiography reports were collected. All treatment introduced during hospitalization and the procedures performed were collected.
- The COVID status of each patient was systematically assessed at ICCU admission using RT-PCR, following current World Health Organization guidelines.

**eSupplement 2. Definition of main admission diagnosis**

- The medical reasons for admission and the main admission diagnosis were adjudicated by a committee of two experts at the end of the hospitalization in each center.
- Main admission diagnosis was categorized into different subgroups: i) ST elevation myocardial infarction (STEMI), including mechanical complication of acute coronary syndrome; ii) non-ST elevation myocardial infarction (NSTEMI); iii) acute heart failure; iv) myocarditis; v) pericarditis; vi) pulmonary embolism; vii) atrial arrhythmia; viii) ventricular arrhythmia; ix) cardiac conduction abnormalities; x) coronary spasm; xi) Takotsubo; xii) aortic dissection; xiii) spontaneous coronary dissection, xiv) chest pain without identified cardiac cause, and xv) other cardiovascular or non-cardiovascular diagnosis.
- Of note, other cardiovascular diagnoses included: infectious endocarditis, acute hypertensive crisis without heart failure, prosthetic valve dysfunction without heart failure, vagal discomfort or orthostatic hypotension without severe cardiac conduction abnormality detected, and monitoring after electrocution.
- Of note, other non-cardiovascular diagnoses included: gastric ulcer, pancreatitis, acute cholecystitis, anemia, exacerbation of COPD, severe asthma, lung or systemic infection, severe COVID-19, migraine crisis, palpitations, or dyspnea without a cardiovascular diagnosis.

| **Main admission diagnosis** | **Definition** |
| --- | --- |
| **Acute coronary syndrome** | Acute coronary syndrome will be defined by typical angina of ≥ 20 min duration, ECG changes, and a rise in troponin or creatine kinase level above the 99^th^ percentile of the upper reference limit after elimination of the differential diagnosis (myopericarditis, Takotsubo syndrome, Tachyarrhythmias, acute heart failure…).^1^  Acute coronary syndrome will be classified as ST-segment elevation and non ST-segment elevation categories.^2^ |
| **Acute heart failure** | An hospitalization for heart failure (HF) will be defined by symptoms and/or signs of HF with evidence of diastolic or systolic dysfunction by echocardiography and elevated levels of natriuretic peptide (BNP >35 pg/ml and/or NT-proBNP >125 pg/ml).^3^ |
| **Myocarditis, pericarditis, Takotsubo syndrome** | Myocarditis will be defined by chest pain, a rise in troponin or creatine kinase level above the 99 percentile of the upper reference limit and confirmation by cardiovascular magnetic resonance (CMR) using the Lake Louis criteria.^4^  Pericarditis will be defined when two out of the four following criteria are fulfilled: a) chest pain b) pericardial rubs c) ECG changes d) pericardial effusion.  Takotsubo syndrome will be defined using the clinical expert consensus statement on takotsubo syndrome.^5^ |
| **Pulmonary Embolism** | Symptoms of pulmonary embolism (dyspnea, chest pain…) confirmed by imaging tests.^6^ |
| **Acute supraventricular arrythmias** | Symptoms of tachycardia (palpitations, dyspnea…) leading to hospitalization in ICCU and 12-lead ECG confirming the supraventricular arrhythmia |
| **Ventricular arrhythmias** | Symptoms of tachycardia (palpitations, dyspnea…) leading to hospitalization in ICCU and 12-lead ECG confirming sustained ventricular tachycardia. |
| **Other** | The subgroup “other diagnosis” includes all diagnoses not eligible for the above categories, including aortic dissection, coronary spasm, unstable angina, endocarditis, hypertensive emergency, acute chest pain without etiology |

**eSupplement 3.** **Variables used in the feature selection algorithm.**

| **Type of variable** | **Detailed features** |
| --- | --- |
| **Demographics** | Age, gender, BMI. |
| **CV risk factors** | Hypertension, dyslipidemia, diabetes, known CAD, family history of CAD. |
| **Medical history of non-CV disease** | Active cancer, psychiatric history |
| **Environmental factors** | Recreational drug use, alcohol consumption, CO level. |
| **Clinical parameters at admission** | Mean arterial pressure, heart rate, oxygen saturation, Killip class |
| **Biological parameters at admission** | Hemoglobin, creatininemia, troponin peak, NTproBNP |
| **Echocardiographic data** | LVEF, LA dilatation, RV dilatation, LV dilation, LV dysfunction, peak E/e’ ratio, TAPSE. |

*Abbreviations: BMI stands for Body Mass Index, CAD stands for Coronary Artery Disease, LA: Left Atrium, LV: Left Ventricular, LVEF: Left Ventricular Ejection Fraction.*

**eSupplement 4. Baseline characteristics of the training and the testing cohorts (N=459).**

|  | **All patients**  **(N=459)** | **Training cohort**  **(N=322)** | **Testing cohort**  **(N=137)** | **p-value** | |
| --- | --- | --- | --- | --- | --- |
| **Demographic data** |  |  |  |  | |
| Age, years | 68 ± 14 | 68 ± 14 | 69 ± 13 | 0.36 | |
| Males, n (%) | 311 (67.8%) | 220 (68.3%) | 91 (66.4%) | 0.69 | |
| Body mass index, kg/m² | 28 ± 6 | 28 ± 7 | 28 ± 6 | 0.82 | |
|  |  |  |  |  | |
| **Cardiovascular risk factors**, n (%) |  |  |  |  | |
| Diabetes mellitus | 131 (28.5%) | 93 (28.9%) | 38 (27.7%) | 0.80 | |
| Hypertension | 287 (62.5%) | 197 (61.2%) | 90 (65.7%) | 0.36 | |
| Dyslipidemia | 204 (44.4%) | 144 (44.7%) | 60 (43.8%) | 0.85 | |
| Current or previous smoking | 285 (63.1%) | 196 (60.9%) | 89 (65.0%) | 0.62 | |
| COPD | 38 (8.3%) | 26 (8.1%) | 12 (8.8%) | 0.75 | |
|  |  |  |  |  | |
| **History of CV disease, n (%)** |  |  |  |  | |
| Known CAD | 107 (23.3%) | 70 (21.7%) | 37 (27.0%) | 0.15 | |
| Renal failure | 96 (20.9%) | 63 (19.6%) | 33 (24.1%) | 0.27 | |
| History of HF hospitalization | 65 (14.2%) | 42 (13.0%) | 23 (16.8%) | 0.29 | |
|  |  |  |  |  | |
| **Clinical parameters at admission** |  |  |  |  | |
| Mean arterial pressure, mmHg | 98 ± 21 | 98 ± 21 | 98 ± 21 | 0.87 | |
| Heart rate, bpm | 91 ± 27 | 92 ± 26 | 89 ± 30 | 0.19 | |
| Oxygen saturation, % | 96.3 ± 3.6 | 96.2 ± 3.8 | 96.7 ± 3.2 | 0.21 | |
| Killip Class |  |  |  |  | |
| I | 216 (47.1%) | 155 (48.1%) | 61 (44.5%) | 0.47 | |
| II | 169 (36.8%) | 115 (35.7%) | 54 (39.4%) | 0.45 | |
| III | 74 (16.1%) | 52 (16.1%) | 22 (16.1%) | 0.98 | |
|  |  |  |  |  | |
| **Main admission diagnosis** |  |  |  |  | |
| Isolated AHF | 197 (43%) | 138 (43%) | 59 (43%) | 0.91 | |
| Acute coronary syndrome | 135 (29%) | 92 (29%) | 43 (30%) | 0.82 | |
| Conduction abnormalities  /arrhythmia | 39 (9%) | 29 (9%) | 10 (7%) | 0.59 | |
| Pulmonary embolism | 29 (6%) | 22 (7%) | 7 (5%) | 0.62 | |
| Acute myocarditis | 20 (4%) | 13 (4%) | 7 (5%) | 0.73 | |
| Takotsubo syndrome | 9 (2%) | 6 (2%) | 3 (2%) | 0.97 | |
| Coronary dissection | 6 (1%) | 3 (1%) | 3 (2%) | 0.49 | |
| Other CV diagnoses | 24 (6%) | 19 (6%) | 5 (4%) | 0.51 | |
|  |  |  |  |  | |
| **Laboratory results** |  |  |  |  | |
|  |  |  |  |  | |
| Hemoglobin, g/dl | 12.9 ± 2.2 | 12.9 ± 2.2 | 12.8 ± 2.1 | 0.60 | |
| Creatinemia, µmol/l | 118 ± 91 | 115 ± 83 | 123 ± 107 | 0.44 | |
| High‐sensitivity cardiac troponin peak, Ul/l | 384 ± 1990 | 402 ± 2252 | 341 ± 1151 | 0.40 | |
| NTproBNP, pg/ml | 13960 ± 23843 | 18549 ± 28083 | 12008 ± 21544 | **0.034** | |
|  |  |  |  |  | |
| **Echocardiography data** |  |  |  |  | |
| LA dilatation ≥ 32 ml/m², n (%) | 144 (31.4%) | 98 (30.4%) | 46 (33.6%) | 0.50 | |
| Mitral E/A ratio | 1.38 ± 0.81 | 1.40 ± 0.82 | 1.32 ± 0.79 | 0.21 | |
| Mitral E/e' ratio | 10.9 ± 4.6 | 11.0 ± 4.8 | 10.7 ± 4.2 | 0.82 | |
| LVEF, % | 45 ± 16 | 46 ± 16 | 44 ± 16 | 0.31 | |
| LVEDV, ml/m² | 127 ± 59 | 126 ± 58 | 131 ± 62 | 0.51 | |
| LVOT VTI, cm | 18 ± 6 | 18 ± 6 | 18 ± 6 | 0.90 | |
| sPAP, mmHg | 32 ± 16 | 32 ± 16 | 32 ± 16 | 0.70 | |
| RV dilatation, n (%) | 63 (14.4%) | 45 (14.5%) | 18 (14.0%) | 0.87 | |
| TAPSE, mm | 19.1 ± 5.0 | 18.9 ± 5.2 | 19.7 ± 4.6 | 0.06 | |
|  |  |  |  |  | |
| **Heart failure data** |  |  |  |  | |
| Worsening heart failure | 95 (20.7%) | 63 (19.6%) | 32 (23.4%) | 0.35 | |
| Ischemic etiology of heart failure | 212 (46.2%) | 141 (43.8%) | 71 (51.8%) | 0.114 | |
| Type of heart failure |  |  |  |  | **0.025** |
| HFrEF | 155 (33.8%) | 99 (30.7%) | 56 (40.9%) |  |  |
| HFmEF | 87 (19.0%) | 70 (21.7%) | 17 (12.4%) |  |  |
| HFpEF | 217 (47.3%) | 153 (47.5%) | 64 (46.7%) |  |  |
|  |  |  |  |  | |
| **Previous treatment** |  |  |  |  | |
| Aldosterone antagonists | 44 (9.6%) | 30 (9.3%) | 14 (10.2%) | 0.76 | |
| ACEI/ARB, or Entresto | 219 (47.7%) | 152 (47.2%) | 67 (48.9%) | 0.73 | |
| Diuretics | 136 (29.6%) | 93 (28.9%) | 43 (31.4%) | 0.59 | |
|  |  |  |  |  | |
| **Environmental factors** |  |  |  |  | |
| CO, ppm | 4.7 ± 5.3 | 4.6 ± 5.1 | 4.9 ± 5.8 | 0.39 | |
| Alcohol consumption | 223 (50.0%) | 158 (50.3%) | 65 (49.2%) | 0.84 | |
| Recreational drug use, n (%) | 42 (9.2%) | 36 (11.2%) | 6 (4.4%) | **0.021** | |
|  |  |  |  |  | |
| **Outcome, n (%)** |  |  |  |  | |
| In-hospital MAE | 47 (10.2%) | 33 (10.2%) | 14 (10.2%) | 0.99 | |

*Abbreviations as in Table 1.*

**eSupplement 5. Feature selection by LASSO**


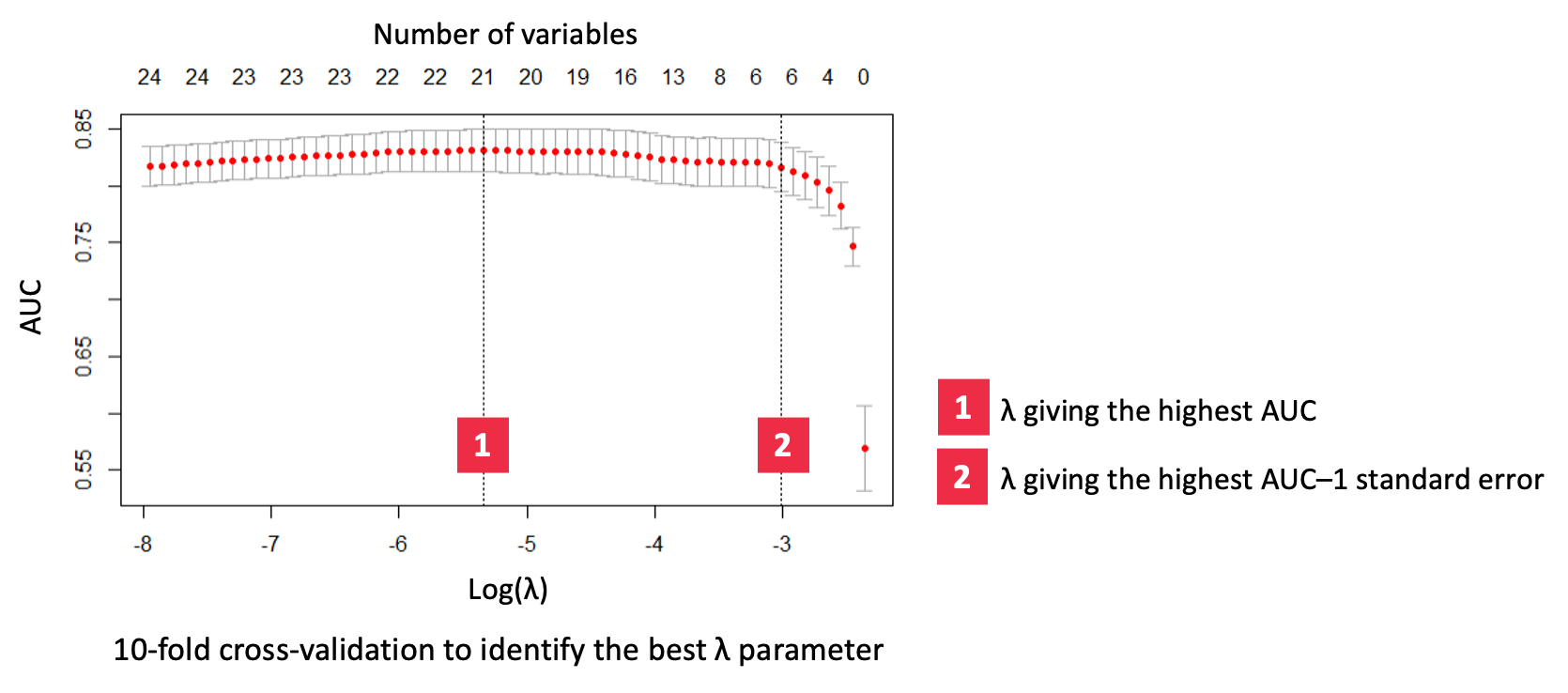


Using the training set (N=322), feature selection was performed using LASSO with 10-fold cross-validation to identify the best regularization parameter λ defined by the λ giving the highest AUC–1 standard error (see Figure). Using the best log(λ) parameter of -3, the best number of variables selected by LASSO was 7, including: left ventricular outflow track velocity time integral (LVOT VTI), peak E/e’ ratio, exhaled CO level, mean arterial pressure, ischemic AHF aetiology, recreational drugs use, and tricuspid annular plane systolic excursion (TAPSE).

**eSupplement 6. Evaluation of the Random Forest performances according to the gender in the testing cohort.**

| **Evaluation metrics** |  | **Males** | **Females** |
| --- | --- | --- | --- |
| ROC AUC |  | 0.82 (0.77-0.87) | 0.83 (0.78-0.89) |
| Precision-recall AUC |  | 0.35 (0.27-0.43) | 0.22 (0.11-0.32) |
| Accuracy |  | 0.93 | 0.85 |
| Cohen’s Kappa |  | 0.63 | 0.38 |
| Sensitivity |  | 0.67 | 0.60 |
| Specificity |  | 0.96 | 0.88 |
| Precision |  | 0.67 | 0.38 |
| F1 score |  | 0.67 | 0.46 |
| Brier score |  | 0.07 | 0.07 |

*Abbreviations: AUC: area under curve; ROC: receiver operating characteristic; LASSO: least absolute shrinkage and selection operator; XGBoost: Extreme gradient boosting.*

**eSupplement 7. Performance of machine learning models for predicting all-cause death.**

| **Evaluation metrics** |  | **Logistic regression** | **Random Forest** |
| --- | --- | --- | --- |
| AUROC |  | 0.76 (0.68-0.80) | 0.81 (0.75-0.88) |
| PRAUC |  | 0.32 (0.25-0.45) | 0.46 (0.34-0.52) |
| Accuracy |  | 0.86 | 0.90 |
| Cohen’s Kappa |  | 0.20 | 0.50 |
| Sensitivity |  | 0.35 | 0.49 |
| Specificity |  | 0.90 | 0.94 |
| Precision |  | 0.35 | 0.60 |
| F1 score |  | 0.35 | 0.56 |
| Brier score |  | 0.08 | 0.07 |

Evaluation of the performance of logistic regression, and Random Forest.

*Abbreviations: AUC: area under curve; ROC: receiver operating characteristic.*

**eSupplement 8. Calibration curve for the ML-model in the testing cohort.**


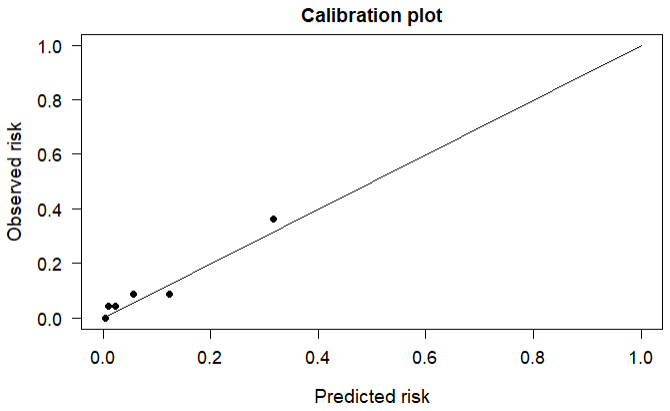


The calibration plot shows the relationship between the observed and predicted proportion of events, grouped by level of risk in the testing cohort. The Random Forest model showed good calibration with the observed in-hospital MAEs. Of note, the Hosmer and Lemeshow goodness-of-fit test showed no significant difference between the proportions of observed and expected predicted in-hospital MAEs (p-value=0.91).

*MAE: major adverse events*

**eReferences**

1. Collet JP, Thiele H, Barbato E, et al. 2020 ESC Guidelines for the management of acute coronary syndromes in patients presenting without persistent ST-segment elevation. *Eur Heart J*. 2021;42(14):1289-1367. doi:10.1093/eurheartj/ehaa575

2. Ibanez B, James S, Agewall S, et al. 2017 ESC Guidelines for the management of acute myocardial infarction in patients presenting with ST-segment elevation: The Task Force for the management of acute myocardial infarction in patients presenting with ST-segment elevation of the European Society of Cardiology (ESC). *Eur Heart J*. 2018;39(2):119-177. doi:10.1093/eurheartj/ehx393

3. McDonagh TA, Metra M, Adamo M, et al. 2021 ESC Guidelines for the diagnosis and treatment of acute and chronic heart failure. *Eur Heart J*. 2021:ehab368. doi:10.1093/eurheartj/ehab368

4. Friedrich MG, Sechtem U, Schulz-Menger J, et al. Cardiovascular magnetic resonance in myocarditis: A JACC White Paper. *J Am Coll Cardiol*. 2009;53(17):1475-1487. doi:10.1016/j.jacc.2009.02.007

5. Ghadri JR, Wittstein IS, Prasad A, et al. International Expert Consensus Document on Takotsubo Syndrome (Part II): Diagnostic Workup, Outcome, and Management. *Eur Heart J*. 2018;39(22):2047-2062. doi:10.1093/eurheartj/ehy077

6. Konstantinides SV, Meyer G, Becattini C, et al. 2019 ESC Guidelines for the diagnosis and management of acute pulmonary embolism developed in collaboration with the European Respiratory Society (ERS). *Eur Heart J*. 2020;41(4):543-603. doi:10.1093/eurheartj/ehz405
